# Pulmonary Valve Papillary Fibroelastoma: A Rare Source of Pulmonary Embolism

**DOI:** 10.7759/cureus.99618

**Published:** 2025-12-19

**Authors:** Clara L Voltarelli, Talita Beithum Ribeiro Mialski, Laura Brandão Proença, João Vitor Firmino, Daniel Hideki Tashima, Kirill Alexandrovitch Nikolin Larkin, Anais de Oliveira Werneck Capistrano, Ana Karyn Ehrenfried Freitas, Jennifer Klassen Boeing, Bruna Erbano

**Affiliations:** 1 Internal Medicine, Hospital Universitário Cajuru, Curitiba, BRA; 2 Cardiology, Universidade Federal do Paraná, Curitiba, BRA; 3 Internal Medicine, Complexo do Hospital de Clínicas da Universidade Federal do Paraná, Curitiba, BRA; 4 Internal Medicine, UniCesumar, Maringá, BRA; 5 Internal Medicine, Faculdade Pequeno Príncipe, Curitiba, BRA

**Keywords:** acute pulmonary embolism, cardiac papillary fibroelastoma, cardiac tumor in adults, echocardiography, pulmonary valve

## Abstract

Papillary fibroelastoma (PFE) is the second most common benign primary cardiac tumor, usually affecting left-sided valves. Pulmonary valve (PV) involvement is extremely rare, representing less than 8% of all PFEs. Although often asymptomatic, PFEs can present with embolic events. We report a 60-year-old man admitted with acute dyspnea, orthopnea, and lower-limb edema. Transthoracic echocardiography revealed severe left ventricular systolic dysfunction (ejection fraction: 15%), severe functional mitral regurgitation, and a mobile echogenic mass (1.0 × 0.8 cm) attached to the arterial surface of the PV consistent with a PFE. Chest computerized tomography (CT) confirmed bilateral segmental pulmonary emboli with infarctions and pulmonary artery dilation. Coronary angiography excluded ischemic disease. Given the patient’s new-onset heart failure with reduced ejection fraction and high operative risk, the heart team opted for initial anticoagulation and optimized heart failure therapy, postponing surgical resection until stabilization. PV PFE is a rare but potentially fatal cause of pulmonary embolism. Multimodality imaging is crucial for diagnosis, and individualized treatment is required to balance embolic risk and surgical mortality.

## Introduction

Primary cardiac tumors are exceedingly rare, with an estimated prevalence ranging between 0.001% and 0.03% in autopsy and imaging studies [1,2]. Despite their rarity, their clinical importance lies in the diversity of possible manifestations: from incidental findings to life-threatening complications, such as heart failure, arrhythmias, or embolic events.

Among benign cardiac tumors, papillary fibroelastoma (PFE) is the second most frequent after myxoma, comprising around 10% of all primary cardiac neoplasms [3]. PFEs are histologically characterized by avascular papillary fronds containing elastic and collagen fibers covered by endothelium. They arise from the endocardial surface, most commonly on the aortic or mitral valves, and appear as small, pedunculated, highly mobile, sea-anemone-like structures on echocardiography [4].

Although benign, PFEs are clinically significant because of their embolic potential. They may serve as a nidus for thrombus formation or undergo fragmentation, leading to systemic or pulmonary embolism, stroke, or myocardial infarction, depending on their location [5,6]. Most are asymptomatic and discovered incidentally during echocardiography, but symptomatic cases are often related to embolization or obstruction of intracardiac blood flow [7].

Pulmonary valve involvement is exceptionally rare, accounting for less than 8% of PFEs, and right-sided lesions are typically asymptomatic [3,4]. However, embolization from pulmonary valve PFEs can result in pulmonary infarction, dyspnea, or acute pulmonary embolism. Such cases are often initially misdiagnosed as thromboembolic disease, particularly in patients with coexisting cardiopulmonary conditions [8].

Multimodality imaging, including transthoracic and transesophageal echocardiography, cardiac computed tomography (CT), and magnetic resonance imaging (MRI), plays a critical role in differentiating PFEs from other valvular or intracardiac masses, such as vegetations, thrombi, Lambl’s excrescences, or metastases [9]. Management depends on the tumor’s location, mobility, and size, as well as on the patient’s surgical risk. Surgical excision is generally curative, but conservative management with anticoagulation and serial imaging may be appropriate in asymptomatic right-sided lesions or when surgery poses a prohibitive risk [10].

## Case presentation

A 60-year-old man with a history of hypertension and chronic use of tobacco, cocaine, and marijuana presented with progressive dyspnea (New York Heart Association (NYHA) class III), orthopnea, and lower-limb edema for the past three weeks. He denied fever or weight loss. Upon examination, there was mild bilateral leg edema and bibasilar crackles.

**Figure 1:**
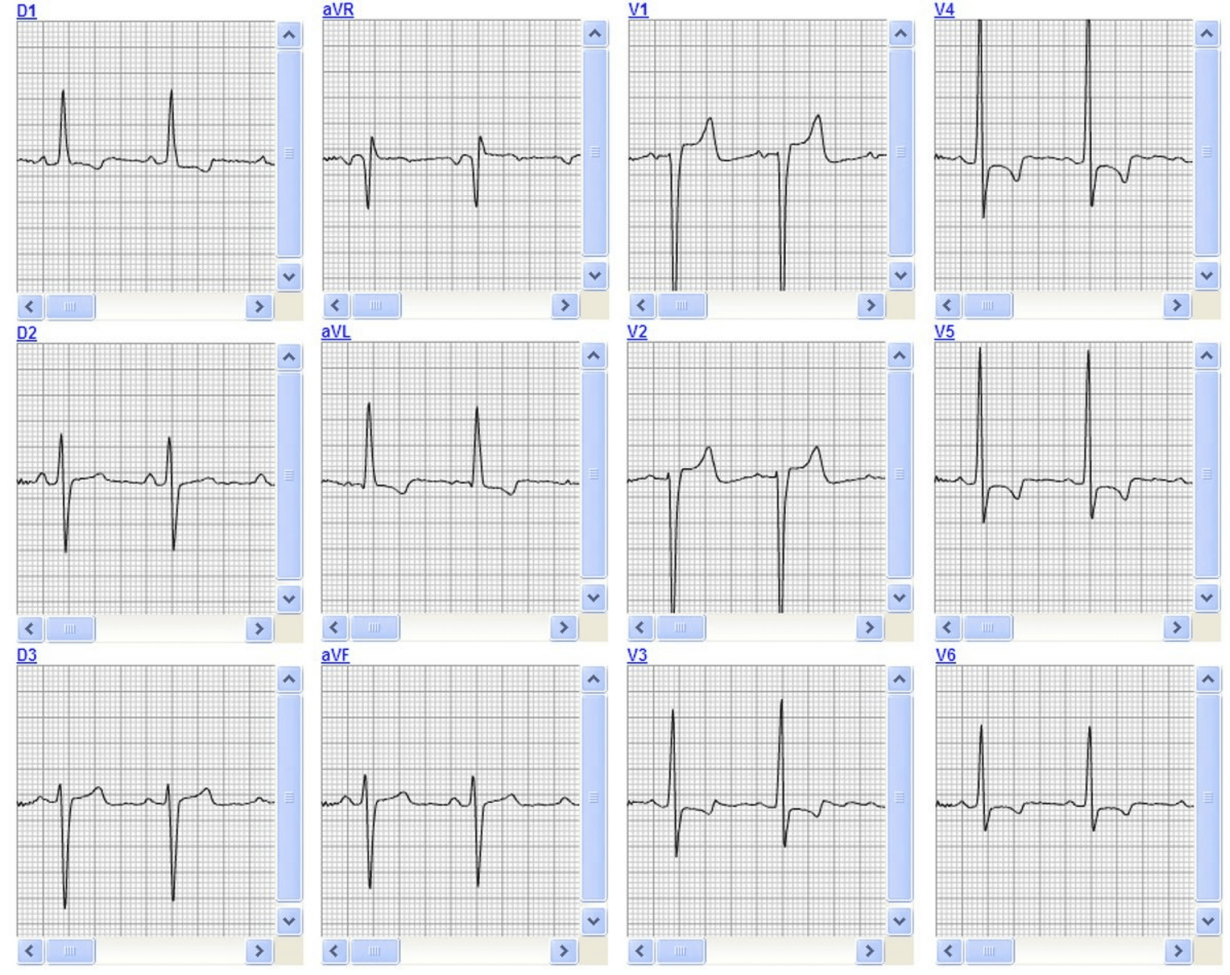
Twelve-lead electrocardiogram demonstrating sinus rhythm, criteria for left ventricular hypertrophy, and left anterior fascicular block.

The electrocardiogram showed sinus rhythm, left ventricular hypertrophy, and left anterior fascicular block (Figure 1). Laboratory testing revealed elevated D-dimer, increased C-reactive protein, and mild renal impairment.

**Figure 2:**
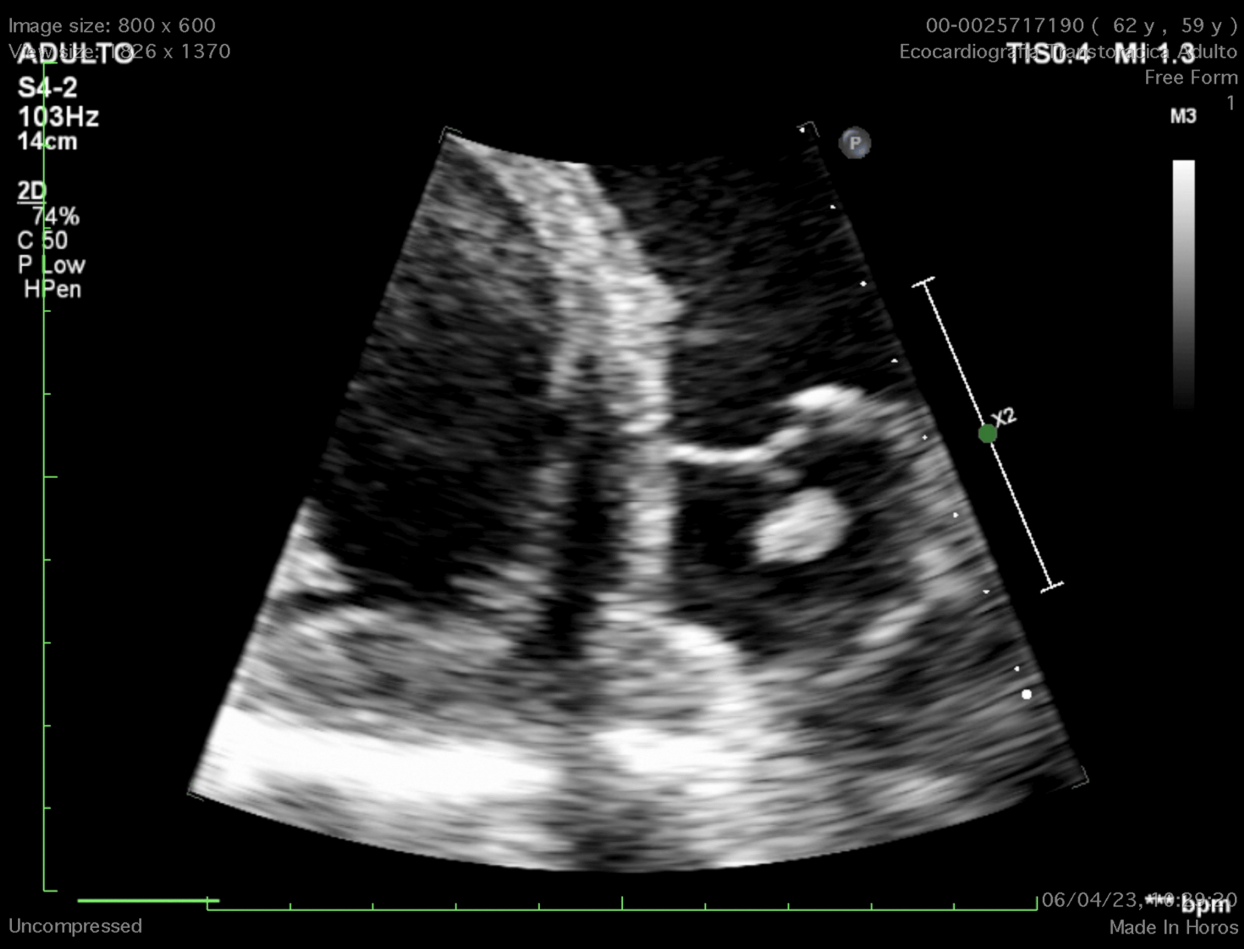
Transthoracic echocardiogram, parasternal short-axis view, showing a papillary fibroelastoma attached to the coaptation surface of the pulmonary valve (zoomed view).

**Figure 3:**
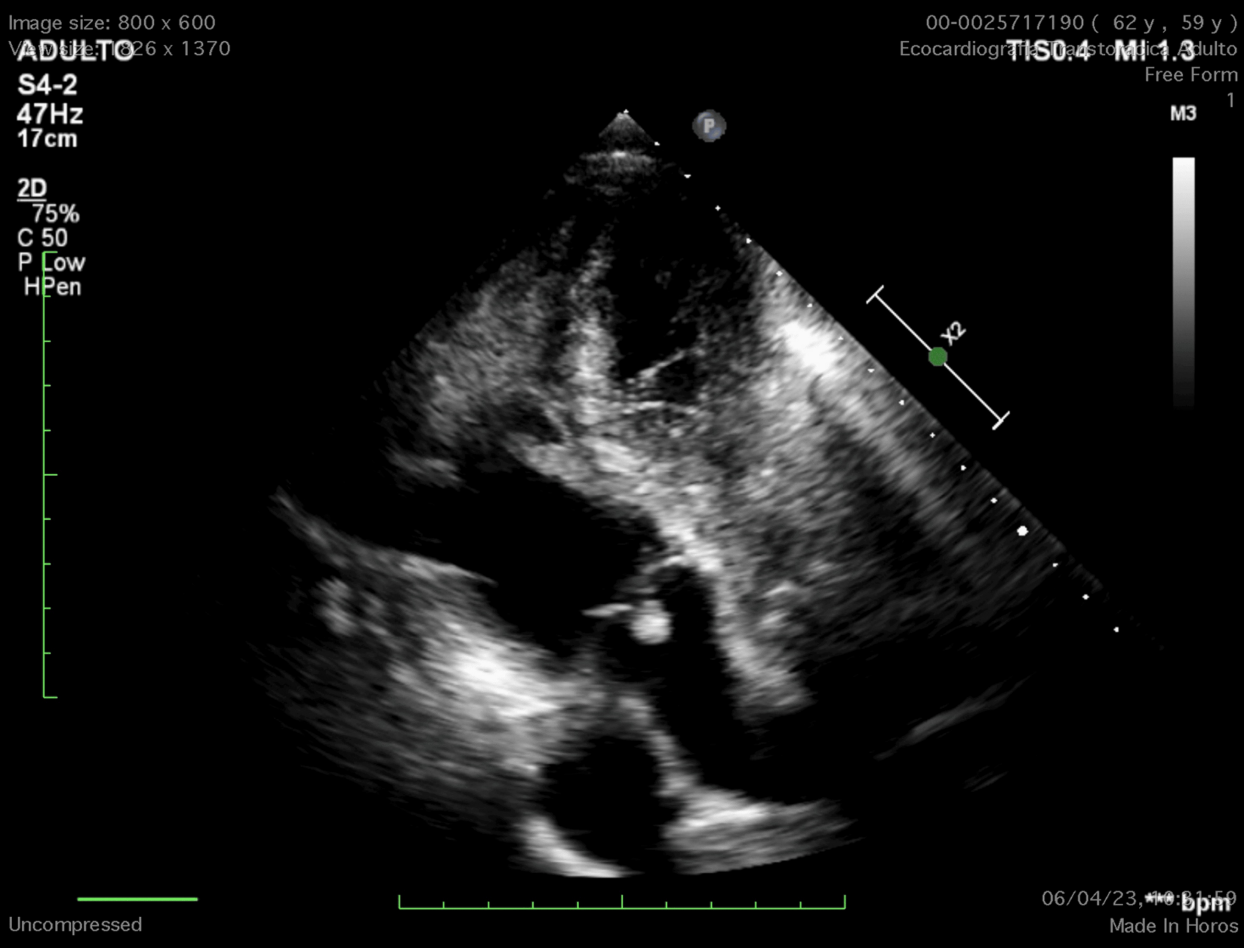
Apical anteriorized view demonstrating the pulmonary valve and the echogenic lesion consistent with papillary fibroelastoma.

Transthoracic echocardiography (TTE) demonstrated severe left ventricular systolic dysfunction with a global ejection fraction of 15% and diffuse hypokinesia, along with severe secondary mitral regurgitation. Additionally, it revealed a hyperechogenic, pedunculated, oval mass (1.0 × 0.8 cm) attached to the arterial surface of the pulmonary valve (Figures 2-3, Videos 1-3). The valve opened normally with only trace regurgitation. Coronary angiography excluded ischemic coronary disease.

**Video 1:** Transthoracic echocardiogram, parasternal short-axis view, showing a papillary fibroelastoma attached to the coaptation surface of the pulmonary valve (zoomed view).

**Video 2:** Transthoracic echocardiogram, parasternal short-axis view, showing the same papillary fibroelastoma attached to the pulmonary valve coaptation surface (standard view).

**Video 3:** Apical four-chamber anteriorized view demonstrating the pulmonary valve and the echogenic lesion consistent with papillary fibroelastoma.

Chest CT angiography confirmed bilateral segmental pulmonary emboli, with peripheral infarctions and pulmonary artery enlargement, findings compatible with embolization originating from the pulmonary valve mass.

A multidisciplinary heart team discussion concluded that the patient had a prohibitive surgical risk, as estimated by the Society of Thoracic Surgeons (STS) score of 17.2%, and was in the early stage of optimized heart failure management. Therefore, a conservative approach was chosen, consisting of guideline-directed medical therapy for heart failure (carvedilol, enalapril, spironolactone, and furosemide). Angiotensin-converting enzyme inhibitor therapy was selected during the initial phase of management in light of the acute clinical setting and renal dysfunction, while angiotensin receptor-neprilysin inhibitor and sodium-glucose cotransporter-2 inhibitor therapies were deferred pending clinical and renal stabilization.

Surgical resection was deferred until clinical stabilization. Despite initial improvement and hospital discharge, the patient unfortunately died suddenly three weeks later, most likely from a cardiac arrhythmia or embolic event.

## Discussion

PFEs are benign endocardial tumors that, despite their histological innocuousness, carry substantial clinical implications due to their high embolic potential. They are typically discovered incidentally but may cause catastrophic complications depending on size, mobility, and attachment site [1-3].

PFEs account for around 8-10% of all cardiac tumors and primarily involve left-sided valves (aortic > mitral) [3,4]. Histologically, they exhibit avascular papillary fronds with a central fibroelastic core and an endothelial covering, resembling sea anemones on echocardiography [5]. Pulmonary valve PFEs are particularly rare, representing less than 1% of all cardiac tumors [3].

Their origin remains debated. The most accepted hypothesis is that they arise from microtrauma-induced endocardial proliferation, leading to fibrin deposition and endothelialization, a process similar to organizing thrombus formation [4,5].

Right-sided PFEs are often asymptomatic but can cause pulmonary embolism when fragments detach, as seen in this case [7]. Left-sided PFEs may cause systemic embolization (stroke or myocardial infarction) [4,5].

Diagnostic imaging plays a central role in the evaluation of PFEs. Transthoracic echocardiography is the primary diagnostic tool, allowing the detection of mobile, pedunculated cardiac masses.

When further anatomical detail is required, transesophageal echocardiography provides superior spatial resolution, particularly for small or valve-adherent lesions. Cardiac CT and MRI complement echocardiography by defining the tumor’s composition, vascularity, and anatomical relationships, which are essential for differentiating fibroelastomas from thrombi or vegetations [8,9]. With current multidetector-row CT technology, even small and highly mobile PFEs may be detected, particularly when a pedunculated stalk is visualized, facilitating differentiation from thrombus [10]. In selected cases, fluorodeoxyglucose positron emission tomography (FDG-PET) may also assist in distinguishing benign from malignant cardiac lesions, although PFEs typically exhibit minimal metabolic uptake [9].

The differential diagnosis of a valvular mass detected on echocardiography includes infective endocarditis, thrombus, Lambl’s excrescences, nonbacterial thrombotic endocarditis (Libman-Sacks), and cardiac myxoma [3-5,8,9]. Infective vegetations typically present as irregular, heterogeneous masses associated with systemic signs of infection and positive blood cultures, features that were absent in this case [8,9]. Thrombi generally lack a pedunculated attachment and often demonstrate variable echogenicity and reduced mobility compared with PFEs [4,5]. Lambl’s excrescences are usually thin, filamentous structures arising along the line of valve closure rather than well-circumscribed nodular lesions [4]. Libman-Sacks endocarditis, classically associated with autoimmune disease, produces sessile, wart-like lesions that differ morphologically from the small, pedunculated, highly mobile mass observed in this patient [8,9]. Cardiac myxomas, although the most common primary cardiac tumors, are typically larger and less mobile and most often arise from the atrial septum rather than from valvular surfaces, making this diagnosis unlikely in the present case [3,5]. Collectively, the echocardiographic morphology, clinical context, and negative infectious workup strongly supported the diagnosis of PFE over alternative entities.

Surgical resection is the treatment of choice for symptomatic or left-sided PFEs and for lesions larger than 1 cm with high mobility, given the higher risk of embolic complications and the excellent outcomes associated with surgical excision [11]. Complete resection is generally curative, with reported recurrence rates below 2% [3,9]. For these reasons, early surgical intervention is typically recommended when operative risk is acceptable.

In contrast, right-sided PFEs (particularly those involving the pulmonary valve) may be managed conservatively in carefully selected patients, especially when surgical risk is elevated. Conservative management consists of systemic anticoagulation and close echocardiographic surveillance, provided the lesion remains stable, non-obstructive, and without significant changes in size or mobility [11]. In such cases, periodic echocardiographic follow-up is essential to detect interval growth or increased mobility that might prompt reconsideration of surgical treatment [3,9]. With appropriate patient selection and follow-up, the overall prognosis of PFEs is excellent.

In this case, the decision to defer surgery was guided by the patient’s severe left ventricular dysfunction (LVEF of 15%), which markedly increases perioperative risk. Studies show that patients with reduced ejection fraction face a higher risk of postoperative mortality, and standard risk calculators such as EuroSCORE and STS may underestimate this risk [2,8]. PFEs are not known to cause systolic dysfunction, and no direct causal relationship between the pulmonary valve lesion and the patient’s severe left ventricular impairment could be established. The clinical presentation was therefore more consistent with an underlying cardiomyopathic process rather than a tumor-related effect. In this context, cocaine-induced cardiomyopathy represents a plausible alternative etiology, as chronic cocaine use has been associated with dilated cardiomyopathy and severe systolic dysfunction through mechanisms including myocardial ischemia, direct myocardial toxicity, and neurohormonal activation [2].

The case thus exemplifies the need for heart team-based, individualized decisions weighing embolic potential against operative mortality.

## Conclusions

Pulmonary valve PFE is a rare but clinically significant cause of pulmonary embolism. Multimodality imaging is fundamental for diagnosis and differentiation from thromboembolic disease. In patients with advanced heart failure or elevated surgical risk, conservative management with anticoagulation and close imaging surveillance may be the safest initial approach. Individualized, multidisciplinary assessment remains crucial to optimize outcomes in these uncommon but potentially fatal cases.
